# Inpatient stroke rehabilitation: prediction of clinical outcomes using a machine-learning approach

**DOI:** 10.1186/s12984-020-00704-3

**Published:** 2020-06-10

**Authors:** Yaar Harari, Megan K. O’Brien, Richard L. Lieber, Arun Jayaraman

**Affiliations:** 1 Max Nader Lab for Rehabilitation Technologies and Outcomes Research, Shirley Ryan AbilityLab, 355 E. Erie St., Chicago, IL 60611 USA; 2 Department of Physical Medicine and Rehabilitation, Northwestern University, Chicago, IL 60611 USA; 3 Department of Biomedical Engineering, Northwestern University, Evanston, IL 60208 USA; 4 Shirley Ryan AbilityLab, Chicago, IL 60611 USA

**Keywords:** Physical therapy, Functional Independence measure, Gait, Balance, Lasso regression

## Abstract

**Background:**

In clinical practice, therapists often rely on clinical outcome measures to quantify a patient’s impairment and function. Predicting a patient’s discharge outcome using baseline clinical information may help clinicians design more targeted treatment strategies and better anticipate the patient’s assistive needs and discharge care plan. The objective of this study was to develop predictive models for four standardized clinical outcome measures (Functional Independence Measure, Ten-Meter Walk Test, Six-Minute Walk Test, Berg Balance Scale) during inpatient rehabilitation.

**Methods:**

Fifty stroke survivors admitted to a United States inpatient rehabilitation hospital participated in this study. Predictors chosen for the clinical discharge scores included demographics, stroke characteristics, and scores of clinical tests at admission. We used the Pearson product-moment and Spearman’s rank correlation coefficients to calculate correlations among clinical outcome measures and predictors, a cross-validated Lasso regression to develop predictive equations for discharge scores of each clinical outcome measure, and a Random Forest based permutation analysis to compare the relative importance of the predictors.

**Results:**

The predictive equations explained 70–77% of the variance in discharge scores and resulted in a normalized error of 13–15% for predicting the outcomes of new patients. The most important predictors were clinical test scores at admission. Additional variables that affected the discharge score of at least one clinical outcome were time from stroke onset to rehabilitation admission, age, sex, body mass index, race, and diagnosis of dysphasia or speech impairment.

**Conclusions:**

The models presented in this study could help clinicians and researchers to predict the discharge scores of clinical outcomes for individuals enrolled in an inpatient stroke rehabilitation program that adheres to U.S. Medicare standards.

## Background

Stroke remains one of the leading causes of disability worldwide, with the majority of stroke survivors requiring specialized rehabilitation [1]. Inpatient stroke rehabilitation is a program of medical intervention and targeted therapies, which aims to maximize a patient’s functional recovery and facilitate reintegration into the community [2, 3]. To evaluate progress, clinicians use standardized assessment tools or clinical outcome measures such as the Functional Independence Measure [4] (FIM) for level of disability or the Ten-Meter Walk Test [5] (TMWT) for walking ability. Understanding the factors that affect these outcomes may help clinicians to streamline the treatment plan and efficiently allocate rehabilitation resources [6, 7]. Further, clinicians assess a patient’s functional abilities based on performance in these standardized tests, such as classifying patients as household ambulators or limited community ambulators based on walking speed score from the TMWT [8, 9]. Estimating a patient’s future discharge scores early in a rehabilitation program would help clinicians set realistic rehabilitation goals and anticipate needs for additional care or medical equipment at discharge.

Several studies have investigated predictors of clinical outcomes after acute inpatient stroke rehabilitation [10–15]. Their main focus was to predict individual’s ability to perform activities of daily living, as measured by the FIM and the Barthel Index [16], or to predict walking speed as measured by the TMWT [14]. These studies found that the clinical assessment scored at discharge could be predicted based on patient demographics such as age [10–13, 15] and sex [11], medical information such as the time from stroke onset to rehabilitation admission [11, 13] and the admission score of the predicted outcome [10–14]. However, there are some notable gaps in our knowledge and understanding of these outcomes. Specifically, previous studies have primarily investigated predictors of a single clinical outcome measure, while therapists often use multiple standardized tests to gauge functional abilities. The American Physical Therapy Association highly recommends additional tests [6], including the Berg Balance Scale [17] (BBS), which assesses balance outcomes and fall risk, and the Six-Minute Walk Test [18] (SMWT), which assesses walking endurance and aerobic capacity. Understanding interactions among different clinical outcomes may help identify the tests that provide unique information about specific functional abilities compared to tests that may be redundant or unrelated to those abilities. Second, studies have predicted the discharge score of a clinical outcome using admission scores from a small subset of other clinical outcomes [14, 19]. For example, discharge walking speed has been predicted from admission scores of BBS and the Motor Assessment Scale [20]. Considering additional admission assessments should improve predictive accuracy, while including additional discharge assessments should provide a more comprehensive overview of a patient’s functional outcomes. Finally, previous studies developed predictive models for clinical outcomes using stepwise methods based on the predictors’ significance level (*p*-value). However, the ability of the p-value to determine the importance of predictors and to output the optimal set of predictors is limited, especially for small sample sizes, small ratio of sample size to predictors, and correlated predictors [21–27]. Conversely, certain machine learning approaches aim to reduce model error by selecting a targeted set of predictors based on relative importance [28] and incorporate regularization mechanisms to produce more accurate and generalizable predictions [29].

The objective of this study was to use machine-learning algorithms to develop predictive models for discharge scores of four standardized clinical tests (FIM, TMWT, SMWT, BBS) after inpatient stroke rehabilitation. Potential predictors included patient demographics, stroke characteristics, and the scores of each of the four tests at admission. We also investigated the correlations between the clinical outcomes and the predictors, stated the predictors’ significance level and compared their relative importance in effecting the discharge scores.

## Methods

Fifty individuals with stroke admitted to the Shirley Ryan AbilityLab (formerly, the Rehabilitation Institute of Chicago) for acute inpatient rehabilitation participated in this study. All individuals (or a proxy) provided written informed consent prior to participation. Inclusion criteria were: diagnosis of stroke and admitted to the Shirley Ryan AbilityLab; at least 18 years of age, and able and willing to give consent and follow study procedure directions. Exclusion criteria were: diagnosis of neurodegenerative pathology as a co-morbidity (e.g., Alzheimer’s disease, Parkinson’s disease, etc.); pregnant or nursing; or utilizing a powered, implanted cardiac device for monitoring or supporting heart function (i.e., pacemaker, defibrillator, or LVAD). Medical clearance was obtained from each patient’s primary physician for study participation. The study was approved by the Institutional Review Board of Northwestern University (Chicago, IL; STU00205532) in accordance with federal regulations, university policies and ethical standards regarding research on human subjects.

After consent, and within the first week of admission, a battery of clinical tests – including the TMWT, SMWT, and BBS – was administered by a licensed physical therapist. These tests were performed in a non-standardized order based on the availability of equipment and space in the therapy room. During the inpatient rehabilitation program, patients received, on average, 180 min of therapy per day, five to 6 days a week. Based on the needs of the patient, this time was divided among physical, occupational, and speech-language therapy. This rehabilitation program follows requirements of Medicare, a major health insurance provider, which sets standards for inpatient stroke rehabilitation in the United States [30]. Within a week of discharge from the hospital, the same battery of clinical tests was again administered to determine the clinical outcomes after inpatient rehabilitation. FIM scores at admission and discharge were compiled from individual FIM items recorded in the patient’s electronic medical records in accordance with the Inpatient Rehabilitation Facility Patient Assessment Instrument guidelines (IRF-PAI, regulated by the United States Centers for Medicare & Medicaid Services). As per hospital standards, the FIM was also administered by licensed physical therapists and performed within 72 h of admission and within the 24–48 h window prior to discharge.

Patient demographics and stroke type were obtained from the Electronic Medical Record (EMR). Diagnoses of dysphagia, cognitive-communication deficit, and other speech/language impairments were made by experienced speech/language pathologists in the hospital and also collected from the EMR as additional stroke characteristics. Finally, patients (or their proxies) completed a study intake form regarding lifestyle and education.

### Dependent and independent variables

The dependent variables were the discharge assessment scores of four commonly used clinical tests: FIM, TMWT, SMWT, BBS.

The independent variables (predictors) included demographic information, stroke characteristics, and scores of the clinical tests from the admission assessment. Demographic information included the patient’s sex, age, body mass index (BMI), race, years of education, and pre-stroke activity levels (defining sedentary as less than 3 h of exercise per week, moderately active as 3–6 h of exercise per week, and highly active as greater than 6 h of exercise per week). Stroke characteristics included time from stroke onset to rehabilitation admission, stroke type (hemorrhagic or ischemic), and diagnoses at admission: dysphagia (i.e., difficulty or discomfort in swallowing), cognitive-communication deficit (i.e., frontal lobe disorders), speech impairments (e.g., aphonia, dysphonia or dysarthria), and language impairment (i.e., aphasia). For analysis, these diagnoses were coded as binary variables (present or absent). The clinical tests at admission included the patients’ FIM, TMWT, SMWT, and BBS scores. Patients who could not walk during a given assessment received a score of 0 for the TMWT or SMWT, in accordance with clinical practice guidelines [31] and similar to previous discharge prediction models [14, 32].

### Data analysis

All statistical analyses were performed using Python version 3.7.3. Normality was evaluated for each dependent variable (i.e. FIM, BBS, TMWT and SMWT) using the Shapiro-Wilk test. For normally-distributed variables, correlations among continuous variables were measured using the Pearson product-moment coefficient (r) and among continuous and categorical variables were measured using the Point-biserial coefficient (r_pb_). For non-parametric variables, correlations were measured using the Spearman’s rank correlation coefficient (r_s_). For all procedures, we considered a coefficient value below 0.3 to express a weak correlation, 0.3 to 0.5 to express a moderate correlation and above 0.5 to express a strong correlation, as recommended by Cohen [33]. Significance level (α) was set to 0.05 and was used to determine which predictors significantly affected each clinical outcome score at discharge.

Predictive models for the discharge scores of each clinical outcome were developed using the cross-validated Lasso regression [29]. Lasso regression is a type of linear regression that includes a regularization term. This term penalizes a model based on the number of predictors and the magnitude of their coefficients. Therefore, it encourages the development of simpler models (fewer predictors) and reduces risk of overfitting [34–37]. The relative strength of the regularization is determined by the value of its parameter λ, wherein λ = 0 produces the same coefficients as linear regression and higher values of λ produce sparser models by forcing more coefficients to 0. In this study, we developed the prediction equations and evaluated their performance using a two-stage, nested, leave-one-out cross-validation (LOOCV) procedure [38, 39]. The outer LOOCV stage was used for evaluating the ability of the model to predict the outcome of a new patient, while the inner stage was used to optimize the parameter λ. In each iteration of the outer stage, the data was divided into train and test sets. Then, the train set was sent to the inner stage and divided again for optimizing λ. Using this procedure ensured that the test set would only be used to evaluate the models performance and never be used for development of the model or optimization of the λ parameter. To quantify the goodness-of-fit of each predictive model, we calculated the percentage of variance explained (***R***^**2**^), and Mean Absolute Error (MAE). To evaluate model performance while accounting for the number of predictors, we also computed the adjusted ***R***^**2**^ (${\boldsymbol{R}}_{\boldsymbol{adj}}^{\mathbf{2}}$). To compare model performance across the different dependent variables, we normalized the MAE of each model by the range of observed values (**MAE**_**n**_). To evaluate the model’s ability to predict both patients that experience small recovery and patients that experience large recovery, we used the Spearman’s rank correlation coefficient (r_s_) and calculated the correlation the patient response to therapy (i.e. change in outcome from admission to discharge) and the model’s error.

We applied the permutation importance analysis based on a Random Forest model [28, 40] to measure the relative importance of the independent variables on each clinical outcome score. Relative importance was established from the contribution of the variable to the predictor in reducing the prediction error. The permutation importance analysis assigned an importance score (*IS*) to each variable, ranging from 0 to 1. The relative importance (*RI*) of a predictor (%) was calculated by dividing the predictor’s score by the sum of all the predictors scores, as follows:

1 $$ {RI}_{i,j}={IS}_{i,j}/\sum \limits_{i=1}^n{IS}_{i,j} $$

where *RI*_*i*, *j*_ is the relative importance of predictor *i* to clinical outcome *j*; *IS*_*i*, *j*_ is the importance score of predictor *i* to clinical outcome *j* assigned by the Random Forest model; and *n* is the number of predictors for clinical outcome *j*. Only variables with *RI*_*i*, *j*_ > 0.01 were considered in the analysis.

## Results

Summary statistics of the patient demographics, stroke characteristics, and clinical test scores are presented in Table 1. The scores of all four clinical outcomes measures significantly improved from admission to discharge (*p* < 0.05). On average, from admission to discharge, FIM scores increased by 47.5% (26.6 points), walking speed from TMWT increased by 61.7% (0.29 m/s), walking endurance from SMWT increased by 82% (185 m), and BBS scores increased by 43% (9 points).

**Table 1:** Demographic information, stroke characteristics, and clinical tests of study participants (*N* = 50)

**Numeric variables**			
**Characteristic**	**Mean**	**SD**	**Range**
Age (y)	57.5	14.15	22–86
Height (cm)	172.9	11.3	149.9–195.6
Weight (kg)	81.3	19.9	42.7–118
BMI (kg/m^2^)	27.1	5.76	16.7–44.7
Education (y)	14.9	3.3	6–20
Rehab duration (days)	18.7	10.4	4–57
Time from stroke to admission (days)	18.8	29.6	3–181
**Categorical variables**			
**Characteristic**	**#**	**%**	
Sex			
*Male*	31	62	
*Female*	19	38	
Race			
*White*	24	48	
*African American*	21	42	
*Hispanic*	4	8	
*Asian*	1	2	
Lifestyle			
*Sedentary*	21	42	
*Moderately active*	11	22	
*Highly active*	18	36	
Stroke type			
*Ischemic*	39	78	
*Hemorrhage*	11	22	
Cognitive/Communication diagnosis			
*Yes*	37	74	
*No*	13	26	
Speech diagnosis			
*Yes*	37	74	
*No*	13	26	
Language diagnosis			
*Yes*	10	20	
*No*	40	80	
Dysphagia			
*Yes*	35	70	
*No*	15	30	
**Clinical tests at admission**			
**Test**	**Mean**	**SD**	**Range**
FIM	55.9	19.1	17–98
TMWT (m/s)	0.47	0.49	0–1.55
SMWT (m)	103.34	122.77	0–461
BBS	20.6	16.26	1–55
**Clinical tests at discharge**			
**Test**	**Mean**	**SD**	**Range**
FIM	82.5	19.5	42–120
TMWT (m/s)	0.76	0.61	0–2.06
SMWT (m)	188.38	163.44	0–562
BBS	29.6	17.41	0–56

### Correlations between clinical outcomes

These results show a strong correlation (0.61 < r_s_ < 0.92) among all clinical outcomes both at admission and at discharge (Table 2). The strongest correlation was found between the TMWT and SMWT at admission (r_s_ = 0.92). All correlations were significant (*p* < 0.05) and positive, such that higher scores in one test indicated higher scores in the other tests.

**Table 2:** Correlations between clinical test scores, at admission and discharge

	Admission				Discharge			
	FIM	TMWT	SMWT	BBS	FIM	TMWT	SMWT	BBS
FIM	1				1			
TMWT	0.61	1			0.73	1		
SMWT	0.72	0.92	1		0.77	0.88	1	
BBS	0.80	0.79	0.88	1	0.86	0.77	0.86	1

### Predictors of clinical outcomes at discharge

All clinical outcomes at discharge (FIM, TMWT, SMWT, BBS) were strongly correlated to the scores of the FIM, TMWT, SMWT, and BBS at admission (0.69 < r_s_ < 0.88; *p* < 0.05), meaning that a high score in one clinical test at admission indicated high scores in all clinical tests at discharge. Time from the stroke onset to admission marginally affected the BBS and TMWT (r_s_ = − 0.24; 0.05 < *p* < 0.1), meaning that shorter time from stroke onset to admission indicated improved clinical outcomes at discharge. The FIM score was moderately correlated with the patient’s sex (r_pb_ = 0.3; *p* < 0.05), with females having higher FIM scores at discharge, and with diagnoses of dysphasia at admission (r_pb_ = 0.32; *p* < 0.05), where dysphagia was related to lower FIM scores at discharge. The BBS score was also moderately correlated with diagnoses of dysphasia (r_s_ = 0.38; *p* < 0.05), where dysphagia was related to lower BBS scores at discharge. Finally, the patient’s age significantly affected the BBS score (r_s_ = − 0.32; *p* < 0.05), and marginally affected the SMWT (r_s_ = − 0.26; 0.05 < *p* < 0.1), where younger patients had greater SMWT and BBS scores at discharge.

### Predictive equations for clinical outcomes at discharge

Predictive models for discharge scores of each clinical outcome were developed using cross-validated Lasso regression (Table 3). The resulting models explained 70–77% of the variance in discharge scores, and average normalized error ranged from 10 to 13% for the study participants and 13–15% for new patients. The generalizability of each model was evaluated using a two-staged nested LOOCV procedure, testing its ability to predict scores of patients that did not participate in the model’s development (Table 3). The LOOCV results show that the MAE increased by an average of 19% to predict the outcomes of a new patient in comparison to the prediction error of the study’s participants. For predicting clinical outcomes of new patients, the average error was 9.5 points for the FIM model (range 0–23), 0.3 m/s for the TMWT model (range 0.01–0.9), 80.8 m for the SMWT model (range 7–256), and 7.4 points for the BBS model (range 0–23).

**Table 3:** Predictive models for the discharge clinical outcomes, including coefficients of each predictor and model goodness-of-fit (*R*^2^, ${R}_{adj}^2$, *MAE*, and *MAE*_*n*_)

	Predictive equation for clinical outcomes at discharge	Study patients (***N*** = 50)		New patients (LOOCV)
		***R***^**2**^$\left({\boldsymbol{R}}_{\boldsymbol{adj}}^{\mathbf{2}}\right)$	MAE (MAE_**n**_)	MAE (MAE_**n**_)
FIM	60.14 + 2.23 ∗ *TMWT* + 0.35 ∗ *FIM* + 0.5 ∗ *BBS* − 0.24 ∗ *age* − 0.02 ∗ *TSA* + 0.8 ∗ *EDU* − 1.71 ∗ *LI* − 0.05 ∗ *BMI* − 10.6 ∗ *HM* − 3.76 ∗ *SI*	0.76 (0.70)	7.6 (0.10)	10.2 (0.13)
TMWT (m/s)	−0.16 + 0.7 ∗ *TMWT* + 0.01 ∗ *FIM* − 0.003 ∗ *TSA* + 0.02 ∗ *EDU* + 0.44 ∗ *HIS* − 0.15 ∗ *LI*	0.70 (0.66)	0.26 (0.13)	0.3 (0.15)
SMWT (m)	190.83 + 101.72 ∗ *TMWT* + 1.03 ∗ *BBS* + 0.54 ∗ *SMWT* − 2.21 ∗ *age*	0.70 (0.67)	73.2 (0.13)	80.8 (0.14)
BBS	13.27 + 10.1 ∗ *TMWT* + 0.33 ∗ *FIM* + 0.21 ∗ *BBS* − 0.24 ∗ *age* − 0.08 ∗ *TSA* + 0.42 ∗ *EDU* − 5.57 ∗ *WHT* − 1.96 ∗ *LI*	0.77 (0.73)	6.4 (0.11)	7.4 (0.14)

*MAE* Mean Absolute Error, *TMWT, FIM, BBS, SMWT* clinical test scores at admission, *TSA* time from stroke to admission, *EDU* education in years, *BMI* Body Mass Index. The following variables are binary and receive the value of 1 or 0: *HM* hemorrhagic stroke, *HIS* Hispanic, *WHT* White, *SI* speech impairment, *LI* language impairment. The “New patients” MAE is the averaged error across all left-out subjects during the LOOCV procedure

**Fig. 1:**
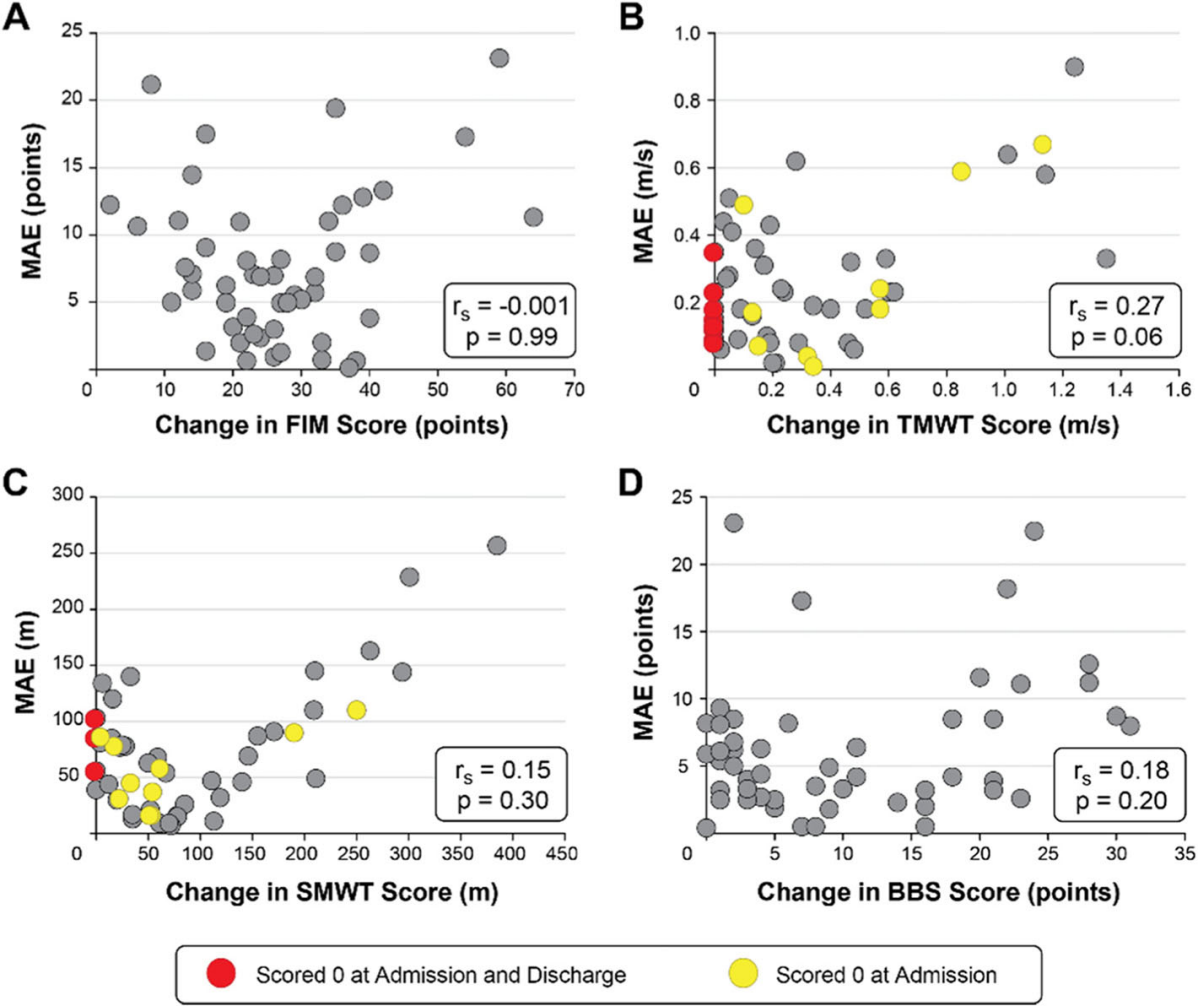
Relationship between patient recovery and model performance. Spearman’s rank correlation between change in clinical score and mean absolute error (MAE) for the (**a**) FIM, (**b**) TMWT, (**c**) SMWT, and (**d**) BBS. Red circles represent patients who scored a 0 at both Admission and Discharge assessments (did not achieve sufficient ambulation ability to complete the test by the end of inpatient rehabilitation); yellow circles represent patients who scored a 0 at Admission but gained sufficient functional ability to complete the test at Discharge

We used Spearman’s coefficient to measure the correlation between the patient response to therapy and the model’s error. The results show a weak (r_s_ ≤ 0.3) and non-significant correlation (*p* > 0.05) for all clinical tests, though there is a trend of greater error for individuals with large change in clinical scores in the TMWT and SMWT (Fig. 1). Patients with a change of 0 in the TMWT and SMWT were unable to complete these tests at both Admission and Discharge due to insufficient ambulation ability. Average MAE for these patients was 0.16 ± 0.10 m/s in the TMWT (*n* = 7; Fig. 1b) and 80.7 ± 23.6 m in the SMWT (*n* = 3; Fig. 1c). On the other hand, some patients were unable to complete these tests at Admission but gained sufficient ambulation ability to attain a score at Discharge. Average MAE for these patients was 0.27 ± 0.25 m/s in the TMWT (*n* = 9; Fig. 1b) and 56.7 ± 32.9 m in the SMWT (*n* = 10; Fig. 1c).

**Fig. 2:**
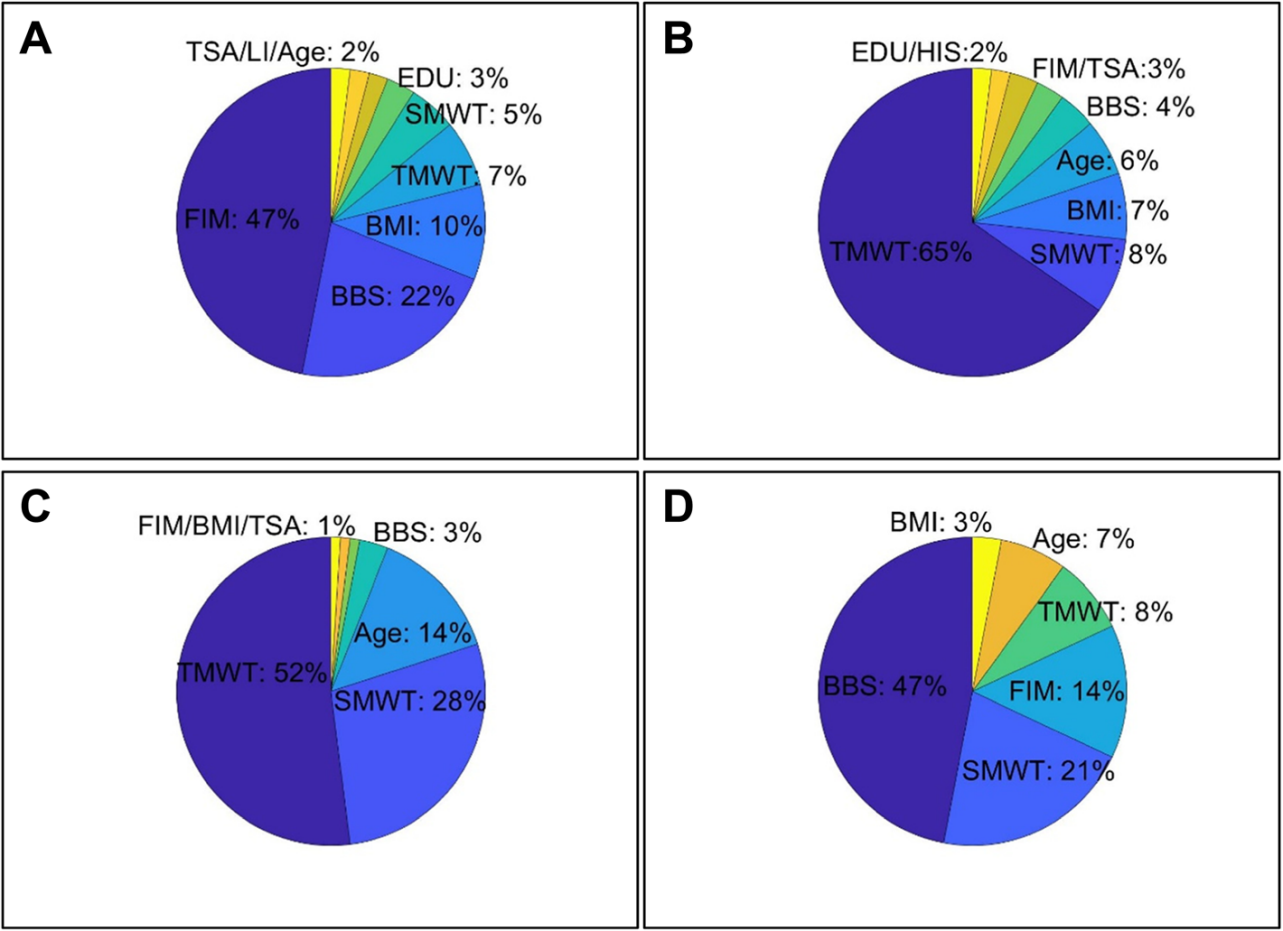
Relative importance of independent variables for discharge clinical outcomes. (**a**) FIM Discharge model; (**b**) TMWT Discharge model; (**c**) SMWT Discharge model; (**d**) BBS Discharge model. Within each model, predictors relating to clinical outcomes tests (FIM, TMWT, SMWT, BBS) are scores from those tests at admission. TSA = time from stroke onset to admission; EDU = education in years; BMI = Body Mass Index; HIS = Hispanic; SI = speech impairment; LI = language impairment; Age = patient’s age in years

The relative importance of the models’ predictors for each clinical outcome at discharge is illustrated graphically in Fig. 2. The most important predictor for the discharge score of the FIM, TMWT, and BBS was their own score at admission. The most important predictor for the SMWT at discharge was the TMWT score at admission. The scores of the clinical tests at admission contributed 80–90% of the relative importance, while demographics and stroke characteristics together contributed the remaining 10–20%.

## Discussion

This study presents a machine learning approach for the prediction of clinical outcomes at discharge after inpatient stroke rehabilitation. The equations developed in this study considered scores of clinical tests at admission, patient demographics, and stroke characteristics as possible predictors, which explained 70–77% of the variance in clinical scores at discharge. The normalized errors for the study’s patients ranged between 0.10–0.13 and for new patients between 0.13–0.15. The permutation analysis found that the most important variables for prediction of the discharge outcomes predictors were the admission scores of the clinical tests. The importance of the scores of clinical test in admission for predicting discharge score was also shown in a previous studies focusing on prediction of FIM [10] and walking speed [14]. Our predictive equations may assist clinicians estimate a trajectory of recovery for their patients during inpatient rehabilitation, using measures that are often available following admission. These results are especially relevant for rehabilitation programs similar to the current study (i.e. following the requirement of Medicare in terms of therapy types and dosage).

We investigated the correlation between the clinical outcomes and found that the TMWT and SMWT were strongly correlated (r_s_ = 0.92), as previously observed by several studies for patients with stroke, spinal cord injury, multiple sclerosis [41–45]. These correlations could explain why only one of the walking tests is included in the FIM, BBS, and TMWT models, since the Lasso regression tends to choose a single variable in a set of correlated predictors [29].

In the current study, apart from the admission scores, additional variables with at least 1% of relative importance for at least one clinical outcome included the time from stroke onset to admission, age, BMI, race, education, dysphasia, and language impairment. Each of these predictors was found to affect clinical outcomes in at least one previous study [10, 13, 14, 46, 47]. The contribution of the current study is in providing a more comprehensive investigation of the clinical tests and set of predictors, in which we found that the relative importance of these variables was much smaller (10–20%) than the importance of the scores of clinical tests at admission (80–90%).

The predictive equation for the FIM discharge score explained 76% of the variance. This model explained more variance than the models presented in all previous studies for predicting FIM at discharge [9, 13, 48], except Ferriero et al. [48] whose model explained 82% of the variance. However, the model of Ferriero et al. [48] included medical comorbidities and complications, which were not considered in the current study. The TMWT discharge predictive equation in the current study explained 70% of the variance, outperforming previous models [15, 19] except for Bland et al. [14] whose model explained 81% of the variance. The model in Bland et al. [14] might have explained more variance because it considered the FIM walk item, which focuses more on elements affecting gait velocity compared to the total FIM score used in the current study. To the best of our knowledge, the current study is the first to develop predictive models for the BBS or SMWT values at discharge.

We applied a machine learning approach to develop predictive models of clinical outcomes at hospital discharge (using cross-validated Lasso regression). Previous studies that predicted discharge scores of clinical outcomes used the *p*-value as a criterion for determining relative importance or selecting features [11, 13, 14, 19]. However, this criterion is prone to overfitting and may not select the most important features, especially in cases where the predictors are strongly correlated [21–27]. In the current study, the feature selection process was performed using the cross-validated Lasso regression, which includes a regularization mechanism (L1) to reduce the risk of overfitting. Since Lasso regression may rule out important variables due to co-linearity with other variables, we investigated the relative importance of the independent variables using permutation importance analysis considering all independent variables. The importance of each variable was evaluated by its ability to reduce error of the Random Forest model which provides a more comprehensive, non-linear, analysis of the relative contributions of each variable to the clinical outcome.

The ability to predict clinical outcomes during stroke rehabilitation remains a meaningful yet challenging task. Clinical test scores at discharge are informative when assessing the patient’s level of independence, ambulation, and risk of falling. Forecasting a patient’s discharge scores early in a rehabilitation program can help clinicians, patients, families, and insurance companies better prepare for the patient’s care needs after leaving the hospital (e.g., to plan discharge location such as skilled nursing facility or home, to estimate the level of assistance the patient will require, to order equipment such as a wheelchair or orthosis, or to evaluate the expected medical costs or insurance coverage). One of the ongoing disputes in the field is the “proportional recovery” rule in stroke recovery [49–52]. Assuming that most stroke patients follow the rule and recover approximately 70% of their functional loss, many studies have developed prediction models of stroke recovery based on admission data [51]. However, recent work has raised important questions regarding the validity of the proportional recovery rule, citing conditions for which models based on this rule might by over-optimistic [49, 50, 52]. In the current study, we tried to avoid this potential pitfall by directly predicting the scores of clinical outcomes at discharge instead of the relative changes in those scores. We acknowledge that our *R*^2^ results might be over-optimistic and thus base our claims on the MAE results. Our models did not identify non-responders in the TMWT and SMWT (individuals who did not attain sufficient ambulation ability to complete these tests by hospital discharge), which is an important area of improvement for clinical prediction models.

Predicting clinical outcomes in the time of admission has been shown to improve therapy efficiency, increasing therapists’ confidence and help to prepare for a probable discharge location [51, 53, 54]. However, the type of rehabilitation program or engagement of the patient could also affect the discharge outcomes. The rehabilitation program in this study is based on the requirements of Medicare, which drives the inpatient rehabilitation structure in the United States, and is expected to be similar to other national inpatient programs. Therefore, the results of this study should be relevant for other U.S. hospitals as well. Future work should consider including objective measures of the rehabilitation program and even measures of patient attitude or engagement during the rehabilitation process in order to further refine the model predictions and improve generalization to alternative rehabilitation programs.

Standard clinical tests alone may not have the prognostic resolution to determine later functional ability. Wearable sensors are an emerging technology that can allow precise, fine-scale measurement of biomechanical and physiological markers during rehabilitation [7, 55]. Such technologies may improve prediction of clinical outcomes by capturing objective, high-resolution data signatures of post-stroke impairment and informing efficient, patient-specific rehabilitation strategies [53]. However, because a sensor-based approach is still in a preliminary research phase and not yet readily available in clinical settings, the models presented in the current study could provide a practical, accessible tool for clinicians to estimate a patient’s recovery trajectory during inpatient rehabilitation.

### Limitations

This study included a relatively small sample size of 50 patients from a single inpatient rehabilitation hospital, which may result in bias, overfitting, and limitations for generalization to other populations. To minimize the effect of small sample size and minimize potential for overfitting, we used Lasso regression [34–37]. Furthermore, the patients who participated in this study had a wide range of demographic characteristics and impairments at admission (Table 1), suggesting that there is moderate variation in the sample for generalization to new patients. Nevertheless, future research could expand the current study by predicting clinical outcomes using a larger sample size from different rehabilitation settings to increase generalizability. The current study included the four clinical outcomes which are highly recommended for evaluation of inpatient stroke rehabilitation by the American Physical Therapy Association [6]. However additional recommended measures could include outcomes such as the Fugl-Meyer Assessment [56] and the Dynamic Gait Index [57], and future research could focus on their prediction.

## Conclusions

We investigated the factors affecting clinical outcomes during inpatient stroke rehabilitation and developed predictive models for their scores at discharge.

All the measured outcomes (FIM, TMWT, SMWT, BBS) were strongly correlated with each other; with the highest correlation found between the TMWT and SMWT (r_s_ = 0.92). The SMWT was not inserted to the model as a predictor for the FIM, BBS or TMWT. Therefore, while the SMWT contributes unique information regarding the patient walking endurance, it might have redundancy with the TMWT for predicting the walking speed (TMWT), balance (BBS) and overall disability (FIM).

The most influential factors for the outcomes scores at discharge were the scores of the clinical test at admission. Therefore, even if a clinicians use only one clinical outcome in their evaluation (e.g. FIM), we recommend to perform additional clinical tests at admission and use their scores as predictors.

The machine learning approach used in this study resulted in the development of predictive models with relatively high percentage of explained variance in comparison to previous studies. Since this approach aims to avoid overfitting, we think these models could be used for other patients as well.

## Data Availability

De-identified data are available from the authors upon reasonable request.

## Abbreviations

FIM: Functional Independence Measure;
TMWT: Ten-Meter Walk Test
BBS: Berg Balance Scale
SMWT: Six-Minute Walk Test
EMR: Electronic Medical Record
BMI: Body mass index
LOOCV: Leave-one-out cross-validation
MAE: Mean Absolute Error

## References

[1] Benjamin EJ, Muntner P, Alonso A, Bittencourt MS, Callaway CW, Carson AP, et al. Heart Disease and Stroke Statistics—2019 Update: A Report From the American Heart Association. Circulation. 139(10):e56–e528. 10.1161/CIR.0000000000000659 30700139

[2] Langhorne P, Bernhardt J, Kwakkel G (2011). Stroke rehabilitation. Lancet..

[3] Quinn T, Paolucci S, Sunnerhagen K, Sivenius J, Walker M, Toni D (2009). Evidence-based stroke rehabilitation: an expanded guidance document from the european stroke organisation (ESO) guidelines for management of ischaemic stroke and transient ischaemic attack 2008. J Rehabil Med.

[4] Hsueh I-P, Lin J-H, Jeng J-S (2002). Comparison of the psychometric characteristics of the functional independence measure, 5 item Barthel index, and 10 item Barthel index in patients with stroke. J Neurol Neurosurg Psychiatry.

[5] Kollen B, Kwakkel G, Lindeman E (2006). Hemiplegic gait after stroke: is measurement of maximum speed required?. Arch Phys Med Rehabil.

[6] Sullivan JE, Crowner BE, Kluding PM, Nichols D, Rose DK, Yoshida R (2013). Outcome measures for individuals with stroke: process and recommendations from the American Physical Therapy Association neurology section task force. Phys Ther.

[7] Smith MC, Barber PA, Stinear CM (2017). The TWIST algorithm predicts time to walking independently after stroke. Neurorehabil Neural Repair.

[8] Stinear C (2010). Prediction of recovery of motor function after stroke. Lancet Neurol.

[9] Smith MC, Byblow WD, Barber PA, Stinear CM (2017). Proportional recovery from lower limb motor impairment after stroke. Stroke Lippincott Williams Wilkins.

[10] Meyer MJ, Pereira S, McClure A, Teasell R, Thind A, Koval J (2015). A systematic review of studies reporting multivariable models to predict functional outcomes after post-stroke inpatient rehabilitation. Disabil Rehabil.

[11] Scrutinio D, Lanzillo B, Guida P, Mastropasqua F, Monitillo V, Pusineri M (2017). Development and validation of a predictive model for functional outcome after stroke rehabilitation the maugeri model. Stroke..

[12] Brown AW, Therneau TM, Schultz BA, Niewczyk PM, Granger CV (2015). Measure of functional Independence dominates discharge outcome prediction after inpatient rehabilitation for stroke. Stroke..

[13] Inouye M, Kishi K, Ikeda Y, Takada M, Katoh J, Iwahashi M (2000). Prediction of functional outcome after stroke rehabilitation. Am J Phys Med Rehabil.

[14] Bland MD, Sturmoski A, Whitson M, Connor LT, Fucetola R, Huskey T (2012). Prediction of discharge walking ability from initial assessment in a stroke inpatient rehabilitation facility population. Arch Phys Med Rehabil.

[15] Goldie PA, Matyas TA, Kinsella GJ, Galea M, Evans OM, Bach TM (1999). Prediction of gait velocity in ambulatory stroke patients during rehabilitation. Arch Phys Med Rehabil.

[16] Quinn TJ, Langhorne P, Stott DJ (2011). Barthel index for stroke trials: development, properties, and application. Stroke..

[17] Blum L, Korner-Bitensky N (2008). Usefulness of the berg balance scale in stroke rehabilitation: a systematic review. Phys Ther.

[18] Chang A, Seale H (2006). Six minute walking test. Aust J Physiother.

[19] Kuys SS, Bew PG, Lynch MR, Morrison G, Brauer SG (2009). Measures of activity limitation on admission to rehabilitation after stroke predict walking speed at discharge: an observational study. Aust J Physiother.

[20] Carr JH, Shepherd RB, Nordholm L, Lynne D (1985). Investigation of a new motor assessment scale for stroke patients. Phys Ther.

[21] Rigby AS (1999). Getting past the statistical referee: moving away from P-values and towards interval estimation. Health Educ Res.

[22] Ranstam J (2012). Why the P-value culture is bad and confidence intervals a better alternative. Osteoarthr Cartil.

[23] Harrell FE (2001). Regression modeling strategies.

[24] Royston P, Sauerbrei W. Multivariable model-building : a pragmatic approach to regression analysis based on fractional polynomials for modelling continuous variables: John Wiley & Sons; 2008.

[25] Heinze G, Dunkler D (2017). Five myths about variable selection. Transpl Int.

[26] Dunkler D, Plischke M, Leffondré K, Heinze G (2014). Augmented backward elimination: a pragmatic and purposeful way to develop statistical models. PLoS One.

[27] Sun G-W, Shook TL, Kay GL (1996). Inappropriate use of bivariable analysis to screen risk factors for use in multivariable analysis. J Clin Epidemiol.

[28] Breiman L (2001). Random Forests. Mach Learn.

[29] Tibshirani R (1996). Regression shrinkage and selection via the lasso. J R Stat Soc B.

[30] Conroy BE, DeJong G, Horn SD (2009). Hospital-based stroke rehabilitation in the United States. Top Stroke Rehabil.

[31] Moore JL, Potter K, Blankshain K, Kaplan SL, O’Dwyer LC, Sullivan JE (2018). A core set of outcome measures for adults with neurologic conditions undergoing rehabilitation. J Neurol Phys Ther.

[32] Hill K, Ellis P, Bernhardt J, Maggs P, Hull S (1997). Balance and mobility outcomes for stroke patients: a comprehensive audit. Aust J Physiother Australian Physiotherapy Association.

[33] Cohen J. Statistical power analysis for the behavioral sciences: Routledge; 2013.

[34] Pavlou M, Ambler G, Seaman S, De Iorio M, Omar RZ (2016). Review and evaluation of penalised regression methods for risk prediction in low-dimensional data with few events. Stat Med.

[35] Majeed YA, Awadalla SS, Patton JL (2018). Regression techniques employing feature selection to predict clinical outcomes in stroke. PLoS One.

[36] Jain D, Singh V (2018). Feature selection and classification systems for chronic disease prediction: a review. Egypt Informatics J.

[37] Pavlou M, Ambler G, Seaman SR, Guttmann O, Elliott P, King M (2015). How to develop a more accurate risk prediction model when there are few events. BMJ..

[38] Stone M (1974). Cross-Validatory choice and assessment of statistical predictions. J R Stat Soc Ser B.

[39] Hastie T, Tibshirani R, Friedman J, Franklin J (2005). The elements of statistical learning: data mining, inference and prediction. Math Intell.

[40] Fisher A, Rudin C, Dominici F (2019). All models are wrong, but many are useful: learning a variable's importance by studying an entire class of prediction models simultaneously. J Mach Learn Res.

[41] Tang A, Sibley KM, Bayley MT, McIlroy WE, Brooks D (2006). Do functional walk tests reflect cardiorespiratory fitness in sub-acute stroke?. J Neuroeng Rehabil.

[42] Dalgas U, Severinsen K, Overgaard K (2012). Relations between 6 minute walking distance and 10 meter walking speed in patients with multiple sclerosis and stroke. Arch Phys Med Rehabil.

[43] Altenburger PA, Dierks TA, Miller KK, Combs SA, Van Puymbroeck M, Schmid AA (2013). Examination of sustained gait speed during extended walking in individuals with chronic stroke. Arch Phys Med Rehabil.

[44] Flansbjer U-B, Holmbäck AM, Downham D, Patten C, Lexell J (2005). Reliability of gait performance tests in men and women with hemiparesis after stroke. J Rehabil Med.

[45] Forrest GF, Hutchinson K, Lorenz DJ, Buehner JJ, VanHiel LR, Sisto SA (2014). Are the 10 meter and 6 minute walk tests redundant in patients with spinal cord injury?. PLoS One.

[46] Hakkennes SJ, Brock K, Hill KD (2011). Selection for inpatient rehabilitation after acute stroke: a systematic review of the literature. Arch Phys Med Rehabil.

[47] Razinia T, Saver JL, Liebeskind DS, Ali LK, Buck B, Ovbiagele B (2007). Body mass index and hospital discharge outcomes after ischemic stroke. Arch Neurol.

[48] Ferriero G, Franchignoni F, Benevolo E, Ottonello M, Scocchi M, Xanthi M (2006). The influence of comorbidities and complications on discharge function in stroke rehabilitation inpatients. Eura Medicophys.

[49] Hope TM, Friston K, Price CJ, Leff AP, Rotshtein P, Bowman H (2019). Recovery after stroke: not so proportional after all?. Brain.

[50] Kundert R, Goldsmith J, Veerbeek JM, Krakauer JW, Luft AR (2019). What the proportional recovery rule is (and is not): methodological and statistical considerations. Neurorehabil Neural Repair.

[51] Stinear CM, Smith M-C, Byblow WD (2019). Prediction tools for stroke rehabilitation. Stroke..

[52] Senesh MR, Reinkensmeyer DJ (2019). Breaking proportional recovery after stroke. Neurorehabil Neural Repair.

[53] Stinear CM, Byblow WD, Ackerley SJ, Barber PA, Smith MC (2017). Predicting recovery potential for individual stroke patients increases rehabilitation efficiency. Stroke..

[54] Brauer SG, Bew PG, Kuys SS, Lynch MR, Morrison G (2008). Prediction of discharge destination after stroke using the motor assessment scale on admission: a prospective, multisite study. Arch Phys Med Rehabil.

[55] Stinear CM (2017). Prediction of motor recovery after stroke: advances in biomarkers. Lancet Neurol.

[56] Sullivan KJ, Tilson JK, Cen SY, Rose DK, Hershberg J, Correa A (2011). Fugl-Meyer assessment of sensorimotor function after stroke: standardized training procedure for clinical practice and clinical trials. Stroke..

[57] Jonsdottir J, Cattaneo D (2007). Reliability and validity of the dynamic gait index in persons with chronic stroke. Arch Phys Med Rehabil.

